# Supplementary material for: Functional genetic characterization of stress tolerance and biofilm formation in Nakaseomyces (Candida) glabrata via a novel CRISPR activation system
Source: mSphere. 2024 Jan 24;9(2):e00761-23. doi: 10.1128/msphere.00761-23 (PMC10900893; doi:10.1128/msphere.00761-23)
Supplement: Supplemental material — Tables S1 to S4. [file msphere.00761-23-s0001.docx]

**Supplemental material**

**Table S1. Strains used in this study.**

| **Name** | **Parental strain** | **Genotype** | **Reference** |
| --- | --- | --- | --- |
| HM100 | CBS138 | ura3::KanX | Muller H, Hennequin C, Gallaud J, Dujon B, Fairhead C. 2008. The asexual yeast Candida glabrata maintains distinct a and alpha haploid mating types. Eukaryot Cell 7:848–858. |
| BG87 | BG2 | ura3::KanX, *his3*Δ | Cormack BP, Falkow S. 1999. Efficient homologous and illegitimate recombination in the opportunistic yeast pathogen Candida glabrata. Genetics 151:979–987. |

**Table S2. Plasmids used in this study.**

| **Name** | **Description** | **Reference** |
| --- | --- | --- |
| pCU-MET3 | Plasmid expression for *N. glabrata*, Ura3+ | Zordan RE, Ren Y, Pan S-J, Rotondo G, De Las Peñas A, Iluore J, Cormack BP. 2013. Expression plasmids for use in Candida glabrata. G3 3:1675–1686. |
| pMET3-dCas9 | Inducible CRISPR-dCas9 for *N. glabrata*, Ura3+ | This study. |
| pPDC1-dCas9 | Constitutive CRISPR-dCas9 for *N. glabrata*, Ura3+ | This study. |
| pCGLM2 (aka pRS712) | Constitutive CRISPRa for *N. glabrata*, Ura3+ | This study. |
| pCU-PDC1 | Plasmid expression for *N. glabrata*, Ura3+ | This study. |

**Table S3. Primers used in this study.**

| **Name** | **Sequence (5’->3’)** | **Localization** |
| --- | --- | --- |
| **Primers for the pCGLM2 and pCU-PDC1 plasmid construction** | | |
| PDC1-F | cgcaattaaccctcactaaagggaacaaaagctggagccAGCATTTTTATACACGTTTT | *PDC1* promoter, Fw |
| PDC1-dCas9-R | caatactatactttttatccatcccgggggatccactatTGTTAATGTTTTTTGGCAAT | *PDC1* promoter, Rv |
| PDC1- R | cggtatcgataagcttgatatcgaattcctgcagcccgggTGTTAATGTTTTTTGGCAAT | *PDC1* promoter, Rv |
| **Primers to amplify crRNA N20 sequences** | | |
| gRNA-extender-F | cgttcgaaacttctccgcagTGAAAGATAAATGATCGGCA | crRNA N20 primers, Fw |
| gRNA-extender-R | gactagccttattttaacttGCTATTTCTAGCTCTAAAAC | crRNA N20 primers, Rv |
| **Primers for RT-qPCRs** | | |
| RT-18S-rRNA-F | TCGGCACCTTACGAGAAATCA | 18S rRNA, Fw |
| RT-18S-rRNA-R | CGACCATACTCCCCCCAGA | 18S rRNA, Rv |
| RT-PDR1-F | TTTGACTCTGTTATGAGCGATTACG | *PDR1*, Fw |
| RT-PDR1-R | TTCGGATTTTTCTGTGACAATGG | *PDR1*, Rv |
| RT-EFG1-F | GAGTGAACCACAACGGAGTTA | *EFG1*, Fw |
| RT-EFG1-R | ATTGCTCTATGCCTTCCTGTC | *EFG1*, Rv |
| RT-SLT2-F | GGACGTTGAGAGACAGACATTTA | *SLT2*, Fw |
| RT-SLT2-R | AACCTGGCAGAACACACTATAC | *SLT2*, Rv |
| RT-STE11-F | GGATCAGACTCCGACTGATAGA | *STE11*, Fw |
| RT-STE11-R | GTGGTGTGGATGGTGGTATT | *STE11*, Rv |

The lowercase letters represent sequences with no homology to template DNA, whereas homologous regions are indicated in uppercase. Abbreviations: Fw, forward; Rv, reverse

**Table S4. Gene fragments used in this study.**

| **Name** | **Sequence (5'--> 3')** | **Length** |
| --- | --- | --- |
| VPR domain | GAAAAAGCGTAAGGTGGATCCTAAGAAAAAGAGAAAGGTTgggcgcgccgaggccagcggttccggacgggctgacgcattggacgattttgatctggatatgctgggaagtgacgccctcgatgattttgaccttgacatgcttggttcggatgcccttgatgactttgacctcgacatgctcggcagtgacgcccttgatgatttcgacctggacatgctgattaactctagaagttccggatctccgaaaaagaaacgcaaagttggtagccagtacctgcccgacaccgacgaccggcaccggatcgaggaaaagcggaagcggacctacgagacattcaagagcatcatgaagaagtcccccttcagcggccccaccgaccctagacctccacctagaagaatcgccgtgcccagcagatccagcgccagcgtgccaaaacctgccccccagccttaccccttcaccagcagcctgagcaccatcaactacgacgagttccctaccatggtgttccccagcggccagatctctcaggcctctgctctggctccagcccctcctcaggtgctgcctcaggctcctgctcctgcaccagctccagccatggtgtctgcactggctcaggcaccagcacccgtgcctgtgctggctcctggacctccacaggctgtggctccaccagcccctaaacctacacaggccggcgagggcacactgtctgaagctctgctgcagctgcagttcgacgacgaggatctgggagccctgctgggaaacagcaccgatcctgccgtgttcaccgacctggccagcgtggacaacagcgagttccagcagctgctgaaccagggcatccctgtggcccctcacaccaccgagcccatgctgatggaataccccgaggccatcacccggctcgtgacaggcgctcagaggcctcctgatccagctcctgcccctctgggagcaccaggcctgcctaatggactgctgtctggcgacgaggacttcagctctatcgccgatatggatttctcagccttgctgggctctggcagcggcagccgggattccagggaagggatgtttttgccgaagcctgaggccggctccgctattagtgacgtgtttgagggccgcgaggtgtgccagccaaaacgaatccggccatttcatcctccaggaagtccatgggccaaccgcccactccccgccagcctcgcaccaacaccaaccggtccagtacatgagccagtcgggtcactgaccccggcaccagtccctcagccactggatccagcgcccgcagtgactcccgaggccagtcacctgttggaggatcccgatgaagagacgagccaggctgtcaaagcccttcgggagatggccgatactgtgattccccagaaggaagaggctgcaatctgtggccaaatggacctttcccatccgcccccaaggggccatctggatgagctgacaaccacacttgagtccatgaccgaggatctgaacctggactcacccctgaccccggaattgaacgagattctggataccttcctgaacgacgagtgcctcttgcatgccatgcatatcagcacaggactgtccatcttcgacacatctctgtttTGATAATCTCTTCTCGAGGGTACCTCATGTAATTAGTTAT | 1,682 bp |

The lowercase letters represent sequences of the VPR tripartite complex, whereas homologous regions to pPDC1-dCas9 are indicated in uppercase.

**Table S5. sgRNAs sequences used in this study.**

| **Name** | **Target gene** | **Position to start codon (bp)** | **Target strand** | **Sequence (5'--> 3')** |
| --- | --- | --- | --- | --- |
| sgRNA-PDR1-1 | *PDR1* | -716 | antisense | GAAAGATAAATGATCGGCA **GCTGGGGTCTTTGTCTGGTTG** GTTTTAGAGCTAGAAATAGC |
| sgRNA-PDR1-2 | *PDR1* | -553 | sense | TGAAAGATAAATGATCGGCA **CCGATGAGCCTCCTATTCCG** GTTTTAGAGCTAGAAATAGC |
| sgRNA-PDR1-3 | *PDR1* | -550 | antisense | GAAAGATAAATGATCGGCA g**CCACGGAATAGGAGGCTCAT** GTTTTAGAGCTAGAAATAGC |
| sgRNA-PDR1-4 | *PDR1* | -470 | sense | TGAAAGATAAATGATCGGCA **TGTGGGTAAAACTAGGTATT** GTTTTAGAGCTAGAAATAGC |
| sgRNA-PDR1-5 | *PDR1* | -385 | antisense | TGAAAGATAAATGATCGGCA **ATTTGGTTGGTATATATGTT** GTTTTAGAGCTAGAAATAGC |
| sgRNA-PDR1-6 | *PDR1* | -191 | sense | TGAAAGATAAATGATCGGCA **AAAGCGTGCCCACACTTGCT** GTTTTAGAGCTAGAAATAGC |
| sgRNA-PDR1-7 | *PDR1* | -164 | antisense | GAAAGATAAATGATCGGCA g**CAGGATAATAACAAGCATAG** GTTTTAGAGCTAGAAATAGC |
| sgRNA-PDR1-8 | *PDR1* | -81 | antisense | GAAAGATAAATGATCGGCA g**CACAATGGCAATACGATATG** GTTTTAGAGCTAGAAATAGC |
| sgRNA-PDR1-9 | *PDR1* | +17 | sense | GAAAGATAAATGATCGGCA g**TACATCAAAATCAAATCCAG** GTTTTAGAGCTAGAAATAGC |
| sgRNA-PDR1-10 | *PDR1* | +36 | antisense | GAAAGATAAATGATCGGCA g**TTCTGTGCTTTGACTTCCCC** GTTTTAGAGCTAGAAATAGC |
| **Name** | **Target gene** | **Position to TSS (bp)** | **Target strand** | **Sequence (5'--> 3')** |
| sgRNA-EFG1-1 | *EFG1* | -462 | antisense | GAAAGATAAATGATCGGCA **aTTCTTGTGTGTATGTGTGTA** GTTTTAGAGCTAGAAATAGC |
| sgRNA-EFG1-2 | *EFG1* | -383 | sense | GAAAGATAAATGATCGGCA **gTGGCGTAGTGTGCATAGTCT** GTTTTAGAGCTAGAAATAGC |
| sgRNA-EFG1-3 | *EFG1* | -238 | antisense | TGAAAGATAAATGATCGGCA **AGCCGTTGTCTGTTGTCCGT** GTTTTAGAGCTAGAAATAGC |
| sgRNA-EFG1-4 | *EFG1* | -204 | sense | TGAAAGATAAATGATCGGCA **AAATGCTAATGCCCCGGAAG** GTTTTAGAGCTAGAAATAGC |
| sgRNA-EFG1-5 | *EFG1* | -190 | antisense | TGAAAGATAAATGATCGGCA **ATTCTATACTCCCACTTCCG** GTTTTAGAGCTAGAAATAGC |
| sgRNA-EFG1-6 | *EFG1* | -139 | sense | TGAAAGATAAATGATCGGCA **ACCCTCGAATGGATAAGCAA** GTTTTAGAGCTAGAAATAGC |
| sgRNA-EFG1-7 | *EFG1* | -105 | sense | TGAAAGATAAATGATCGGCA **GATGAGCCTTGTGAGCAGAG** GTTTTAGAGCTAGAAATAGC |
| sgRNA-EFG1-8 | *EFG1* | -36 | sense | GAAAGATAAATGATCGGCA **gCTTGGGTGCTCTTCCATCAA** GTTTTAGAGCTAGAAATAGC |
| sgRNA-EFG1-9 | *EFG1* | +30 | sense | GAAAGATAAATGATCGGCA **aTGGTGGGAGCAGGGAGTTAC** GTTTTAGAGCTAGAAATAGC |
| sgRNA-EFG1-10 | *EFG1* | +112 | antisense | TGAAAGATAAATGATCGGCA **GGGGGTAAGTATGTGTATCC** GTTTTAGAGCTAGAAATAGC |
| sgRNA-STE11-1 | *STE11* | -247 | sense | TGAAAGATAAATGATCGGCA **ATTTGAGTAGTATAGTGTAG** GTTTTAGAGCTAGAAATAGC |
| sgRNA-STE11-2 | *STE11* | -183 | antisense | GAAAGATAAATGATCGGCA **gTTACTGAAGAATCATGTCTT** GTTTTAGAGCTAGAAATAGC |
| sgRNA-STE11-3 | *STE11* | -144 | antisense | GAAAGATAAATGATCGGCA **gTACGAGATGATGTATAGTAA** GTTTTAGAGCTAGAAATAGC |
| sgRNA-STE11-4 | *STE11* | -96 | antisense | TGAAAGATAAATGATCGGCA **AAACAGCTATAAATAGCCCC** GTTTTAGAGCTAGAAATAGC |
| sgRNA-STE11-5 | *STE11* | -9 | antisense | TGAAAGATAAATGATCGGCA **AACCGATTTGATCTTAAGTA** GTTTTAGAGCTAGAAATAGC |
| sgRNA-SLT2-1 | *SLT2* | -34 | antisense | TGAAAGATAAATGATCGGCA **AATCACTGAAACAAGGCTTC** GTTTTAGAGCTAGAAATAGC |
| sgRNA-SLT2-2 | *SLT2* | -112 | antisense | GAAAGATAAATGATCGGCA **gTCAGTGTGATTGTCCCCCGG** GTTTTAGAGCTAGAAATAGC |
| sgRNA-SLT2-3 | *SLT2* | -191 | sense | TGAAAGATAAATGATCGGCA **AAACATCAGTGGCTGGACGG** GTTTTAGAGCTAGAAATAGC |
| sgRNA-SLT2-4 | *SLT2* | -360 | sense | TGAAAGATAAATGATCGGCA **TATGCCCAATATACTCAATG** GTTTTAGAGCTAGAAATAGC |
| Non-targeting sgRNA | None | N/A | N/A | TGAAAGATAAATGATCGGCA **CAAGTGTCTGAAGAACAACT** GTTTTAGAGCTAGAAATAGC |

Underlined sequences represent bp identical to the CRISPRa plasmid for Gibson assembly cloning and bold sequences N20 crRNA sequences.
